# Network pharmacology-based approach to research the effects and mechanisms of *Salvia Miltiorrhiza* injection against idiopathic pulmonary fibrosis

**DOI:** 10.3389/fmed.2025.1569590

**Published:** 2025-06-24

**Authors:** Liangyu Chen, Haobo Lin, Linmang Qin, Guangfeng Zhang, Peisheng Chen, Zebo Jiang, Pan Xu, Donghui Huang, Xiao Zhang

**Affiliations:** ^1^Faculty of Chinese Medicine, Macau University of Science and Technology, Taipa, Macao SAR, China; ^2^Department of Respiratory and Critical Care Medicine, Zhuhai Hospital of Integrated Traditional Chinese and Western Medicine, Zhuhai, China; ^3^Department of Rheumatology, Guangdong Provincial People’s Hospital, Guangzhou, China; ^4^Guangdong Academy of Medical Sciences, Guangzhou, China; ^5^Southern Medical University, Guangzhou, China; ^6^Department of Rheumatology, The Eighth Affiliated Hospital, Sun Yat-sen University, Shenzhen, China

**Keywords:** *Salvia Miltiorrhiza* injection, idiopathic pulmonary fibrosis, network pharmacology, inflammation, fibroblast

## Abstract

**Background:**

Idiopathic pulmonary fibrosis (IPF) is a progressive, life-threatening lung disease with limited treatment efficacy. *Salvia miltiorrhiza* (SM), a traditional Chinese medicine (TCM), is widely used in Chinese hospitals due to its antithrombotic, anti-inflammatory, and antioxidant properties. SM has also demonstrated potential as an anti-fibrotic agent. This study aims to investigate the therapeutic effects and mechanisms of SM injection in treating IPF.

**Methods:**

Active components and targets of SM were acquired from the Traditional Chinese Medicine Systems Pharmacology (TCMSP) database, while IPF-associated genes were obtained from the DisGeNET database. Venn analysis was applied to intersect SM targets with IPF-associated genes, identifying potential therapeutic targets. A protein–protein interaction (PPI) network of these targets was constructed using the STRING database and visualized with Cytoscape software, where the CytoHubba plug-in was utilized to determine core therapeutic targets. Gene Ontology (GO) and Kyoto Encyclopedia of Genes and Genomes (KEGG) analyses of the core targets were conducted via R language, and molecular docking was performed to predict the binding affinities of active compounds to the core targets. The core targets were further validated through qRT-PCR, Western blot (WB), and ELISA experiments.

**Results:**

70 potential target genes of SM injection for the treatment of IPF were identified, with MMP9, IL-6, and TNF-*α* as the core targets. These core targets were linked to pathways involving inflammation, oxidative stress, and extracellular matrix (ECM) remodeling. *In vitro* experiments indicated that SM injection alleviated pulmonary fibrosis by downregulating MMP9, IL-6, and TNF-*α*.

**Conclusion:**

SM injection may effectively reduce pulmonary fibrosis through multi-target mechanisms, providing a new therapeutic strategy for IPF from the perspective of TCM.

## 1 Introduction

IPF is a chronic, progressive disease that features inflammatory injury, fibroblast activation, extensive accumulation of ECM, and destruction of lung tissue structure (1, 2). Symptoms typically include cough and progressive dyspnea, which can develop into irreversible respiratory failure. If left untreated, the average lifespan of IPF patients ranges from 3 to 5 years after diagnosis (3). Genetic and environmental factors are considered risk factors for IPF, and some drugs, radiation therapies, microbial infections, and other diseases may also be involved in the progression of IPF (4–6).

Though the exact cause of IPF has not been fully understood, research shows that it is mainly driven by abnormal repair of recurrently injured lung epitheliums and dysregulation of lung fibroblasts (2, 4). In addition, the onset of IPF is closely associated with biological processes including coagulation reactions, oxidative stress, inflammation, and immune responses. In particular, the inflammatory immune response and oxidative stress are believed to play critical roles in the pathological mechanism of IPF (4). For instance, some innate immunity cells (e.g., neutrophils and macrophages), adaptive immunity cells (e.g., Th1, Th2, Th17, and B cells), and inflammatory mediators (e.g., interleukin, TGF-*β*, PDGF, and VEGF) have exhibited pro-fibrotic functions during the progression of IPF. These immune cells and inflammatory mediators directly or indirectly promote fibrosis by inducing lung epithelial cell injury, activating fibroblasts, and enhancing ECM deposition (4, 7).

In terms of oxidative stress, the overproduction of reactive oxygen species (ROS) and the imbalance of antioxidant defense systems are considered key mechanisms in IPF. ROS drives alveolar epithelial cell injury and fibroblast activation through multiple mechanisms, including DNA damage, epigenetic alterations, cellular senescence, dysregulated proteostasis, and mitochondrial dysfunction, thereby exacerbating fibrosis (8). Moreover, there is an interplay between oxidative stress and immune-mediated inflammation: oxidative stress activates inflammatory pathways such as TGF-*β* and NF-κB, while inflammatory immune responses intensify oxidative stress through mechanisms like Nrf2 inhibition (9, 10).

Among these mechanisms, TGF-β stands out as a prominent and crucial trigger. This cytokine exerts its pro-fibrotic effects primarily through the Smad signaling pathway, where Smad2/3 proteins are phosphorylated and translocate to the nucleus to regulate the transcription of fibrosis-related genes (11). This signaling cascade subsequently facilitates the epithelial-mesenchymal transition (EMT) and fibroblast-to-myofibroblast differentiation, leading to ECM deposition and the upregulation of fibrosis biomarkers such as *α*-smooth muscle actin (α-SMA) and fibronectin 1 (Fn1) (4, 12, 13). α-SMA enhances the contractile function of myofibroblasts, while Fn1 aids in the remodeling of the ECM, both of which are essential in the process of fibrosis. Consequently, TGF-*β* is commonly employed to culture human lung fibroblasts (HLFs) to create the *in vitro* models of lung fibrosis, and *α*-SMA and Fn1 are frequently used to assess the extent and progression of fibrosis (14–16).

Currently, the well-acknowledged effective treatments for IPF are lung transplantation and anti-fibrotic medications, including pirfenidone and nintedanib. However, the high expenses, severe complications, side effects, and other underlying risks of these therapies have contributed to the high mortality of this disease (17, 18).

SM, usually referred to as “Danshen” in Chinese, is an herb widely used in TCM. SM is rich in various active compounds, including lipophilic tanshinones and water-soluble phenolic acids. These ingredients have demonstrated diverse biological actions, such as anti-inflammatory, antioxidative, and antithrombotic effects (19). Studies have also indicated that some components of SM have anti-fibrotic pharmacological mechanisms. For example, tanshinone IIA alleviates pulmonary fibrosis by modulating inflammation and oxidative stress, suppressing alveolar epithelial cell pyroptosis, and ameliorating energy metabolism dysfunction (20–24). Danshensu (Salvianic acid A) ameliorates lung fibrosis by preventing fibroblast-to-myofibroblast transition (25). Salvianolic acid A mitigates pulmonary fibrosis by inhibiting fibroblast activation and promoting apoptosis (26). Salvianolic acid B exerts therapeutic effects against pulmonary fibrosis through inhibiting fibroblast activation, reducing oxidative stress, regulating inflammatory cytokines, and attenuating alveolar epithelial cell senescence (22, 27, 28). Cryptotanshinone exhibits anti-fibrotic properties by reversing EMT, modulating macrophage polarization, and targeting inflammatory pathways (29–31). Nevertheless, pharmaceutical development on these individual components may encounter obstacles such as high research and development costs, unknown safety, a lack of standardized doses and forms, and limited clinical evidence. On the contrary, as a commonly used TCM injection in Chinese hospitals, SM injection is easily accessible and cost-effective. It also has a well-defined safe dosage, a consistent clinical safety profile, and predictable side effects (32–34). Besides, SM injection may exert a stronger effect than individual components, for it may contain more underlying anti-fibrotic ingredients, and the components may work together to more effectively protect against pulmonary fibrosis. Therefore, exploring the effects and mechanisms of SM injection on IPF carries greater practical and clinical significance.

In the past, due to technological limitations, the study of Chinese herbal medicine faced various challenges, including the complexity of multiple components and their potential synergistic effects, making the exploration of therapeutic mechanisms complicated and difficult (35). However, with the development of technologies such as network pharmacology and bioinformatics, the analysis of the multiple ingredients in Chinese medicine has now become possible. This progress has been greatly facilitated by network pharmacology, which studies the complex interactions between drugs and biological networks to understand drug effects and therapeutic targets. Rather than focusing on a single component, target, or pathway, this approach integrates bioinformatics and system biology to analyze how pharmaceuticals impact biological networks and to build the multilayered relationships between components, targets, and diseases (36). Utilizing network pharmacology analysis, our study intends to explore the multi-target and multi-pathway mechanisms of SM injection in treating IPF, which may help to identify new drug targets and pathways, develop innovative medicine, and offer new insights for IPF treatment.

## 2 Materials and methods

### 2.1 Collection of compounds and target genes associated with SM injection

We obtained the chemical constituents of SM injection from the TCMSP database (version: 2.3), which can be accessed at https://old.tcmsp-e.com/tcmsp.php. This database offers 12 pharmacokinetic properties, including oral bioavailability (OB), drug half-life (HL), drug-likeness (DL), and blood–brain barrier (BBB) permeability (37). Although the well-established selection criteria for active compounds in Chinese herbal medicine typically require DL ≥ 0.18 and OB ≥ 30% (38, 39), we selected compounds with DL ≥ 0.18 as the analysis objects, considering that SM injection is administered intravenously in clinical practice. Additionally, we included danshensu (salvianic acid A) and protocatechualdehyde in our analysis, despite their DL values being less than 0.18. This decision is based on studies indicating that danshensu and protocatechualdehyde are the two major hydrophilic constituents of SM with remarkable pharmacological effects (40, 41). It’s also important to note that “*Salvia Miltiorrhiza*” is referred to as “Radix Salviae” in the TCMSP database due to the traditional use of its root in Chinese medicine. The targets for each selected compound were also collected from the TCMSP database and subsequently converted to their official gene symbols using the UniProt database.^1^ The redundant targets were merged.

### 2.2 Establishment of compound-target-pathway network

The targets of the selected compounds were imported into the Database for Annotation, Visualization, and Integrated Discovery (DAVID, version: 2021 update, https://david.ncifcrf.gov/) web server for KEGG analysis (42). Next, the selected ingredients of SM, the targets of these ingredients, and the top 50 KEGG pathways associated with the targets were loaded into Cytoscape software to construct a compound-target-pathway network, visualizing the interactions between compounds, targets, and the related pathways.

### 2.3 Identification of IPF-related genes

We utilized “idiopathic pulmonary fibrosis,” “familial idiopathic pulmonary fibrosis,” “exacerbation of idiopathic pulmonary fibrosis,” “chronic idiopathic pulmonary fibrosis,” “idiopathic pulmonary fibrosis (acute form),” “diffuse idiopathic pulmonary fibrosis,” and “idiopathic interstitial pneumonias” as keywords to collect disease-related genes from the DisGeNET database^2^ (version: 24.2). DisGeNET is a comprehensive resource that integrates gene-disease connections from numerous repositories for use in biomedical research and genomics studies (43). The duplicated genes screened among these keywords were consolidated.

### 2.4 Venn diagram

A Venn diagram was utilized to obtain the intersection between IPF-related genes and targets associated with SM injection, which were defined as “potential therapeutic targets for IPF.”

### 2.5 Construction of PPI network and screening of key targets

The intersected target genes were mapped onto the Search Tool for the Retrieval of Interacting Genes (STRING, Version: 12.0, https://string-db.org/) database, with the organism restricted to “*Homo sapiens*.” The results were then imported into Cytoscape 3.9.1 for PPI network construction. Within the network, the degree values of each node were measured to assess their significance (44, 45), and their relative importance was presented with node colors: red denotes the most critical nodes, while yellow indicates the least critical. Orange represents a medium level of importance, with lighter shades of orange signifying a lower medium importance and darker shades suggesting a higher medium importance, bridging the gap between red and yellow. Key targets within the network were subsequently identified by CytoHubba, a Cytoscape plugin used to filter key genes (45). Six algorithms were applied for screening, including degree, radiality, closeness, stress, edge percolated component (EPC), and maximal neighborhood component (MNC) (15, 46, 47). The intersection of the top 5 filtered genes from each method was defined as key targets.

### 2.6 Enrichment analyses of key therapeutic targets for IPF

The biological activities and pathways linked to the key targets of SM in treating IPF were investigated through GO and KEGG analyses applying the “ClusterProfiler” R package—a versatile tool within the R language designed for bioinformatics and computational biology, enabling statistical analysis and visualization of gene clusters (48). The utilization of GO analysis was to explore the cellular components (CC), biological processes (BP) and molecular functions (MF), while KEGG analysis was employed to discover the signaling pathways related to the target genes (49, 50). The top 10 enriched categories from GO analysis and the top 5 enriched pathways from KEGG analysis were presented via bar plots and dot plots.

### 2.7 Molecular docking

Molecular docking is a computational technique used in structural biology and drug discovery to simulate and predict the binding mechanisms between small molecules (ligands) and target proteins. It involves simulating the binding between a compound and a target protein to determine the most favorable orientation and conformation of the compound within the protein’s binding site (51). Using the compound-target-pathway network previously constructed (seen in Section 2.2), the significance of compounds was evaluated according to their degree values. The top 15 compounds with the highest degree scores and the key therapeutic targets were chosen for molecular docking studies. To begin, the crystal structure of the target protein was sourced from the PDB database^3^ (52). Secondly, dehydrogenation, energy optimization, amino acid modification, and adjustment of force field parameters were performed using PyMOL^4^ (version: 3.0) and AutoDock Vina^5^ (version: 1.2.0) (53). For the compounds, the structure was acquired from PubChem^6^ (version: 2023 update) (54), then docked with core targets using AutoDock Vina after adding hydrogen and calculating charges. The lowest binding energy for each complex was determined to be optimal. Energies of ≤ − 4.25 kcal/mol, ≤ − 5.0 kcal/mol, and ≤ − 7.0 kcal/mol were indicative of reasonable, good, and strong binding affinity, respectively (44). The top 3 strongest docked complexes for each key target were visualized with PyMOL software.

### 2.8 Extraction and culture of primary HLFs

Lung tissue samples were acquired from patients undergoing lobectomies for lung cancer at the Department of Thoracic Surgery, Guangdong Provincial People’s Hospital. All participating patients had signed written informed consent before their inclusion in the study. These tissues, confirmed as healthy via biopsy and located more than 5 cm from lesion sites, were cut into 1 mm^3^ sections. These pieces were evenly dispersed across culture dishes, which were subsequently inverted and incubated at 37°C with 5% CO_2_. 4 h later, when the tissue pieces had adhered to the bottoms of the dishes, the dishes were gently reverted to their upright position. High-glucose Dulbecco’s Modified Eagle Medium (DMEM, Thermo Fisher, United States), supplemented with 1% penicillin–streptomycin and 20% fetal bovine serum (ZETA LIFE, United States) mixture, was then added. Primary HLFs were isolated as they migrated from the tissues and formed colonies. After 4–6 passages in culture, these patient-derived fibroblasts were utilized for the following studies.

### 2.9 CCK-8 experiment

The CCK-8 assay is known for its rapid and highly sensitive assessment of cell viability in diverse experimental settings, such as cytotoxicity tests, cell proliferation studies, and drug concentration selection. In this study, cell viability was assessed using the CCK-8 kit (Beyotime, China) to evaluate the cytotoxic effects of SM injection on HLFs and determine its optimal experimental concentration. Firstly, the HLF suspensions (2 × 10^4 cells/well) were preincubated in 96-well plates with 100 μL of medium. Once the cells had adhered, they were treated with various concentrations of SM injection (0, 0.625, 1.25, 2.5, 5, 10, 20, 40, 80, and 160 μL/mL) and then incubated at 37°C for 48 h. Afterward, CCK-8 reagent was added and cultured for 30 min. Absorbance at 450 nm was subsequently measured using a microplate reader (Multiskan GO, Thermo, United States). The cell viability under different experimental conditions was calculated based on the absorbance measurements and visualized using a bar plot created with GraphPad Prism 8 software. The IC50 value was also determined from the cell viability data using the same software. Based on the results from the CCK-8 assay, an appropriate concentration of SM injection was selected for further experiments.

### 2.10 Grouping and intervention

Following extraction from patients and subsequent culture for 4–6 passages, the HLFs were seeded into 6-well plates at a density of 3 × 10^5 cells per well and assigned to 3 groups: the control group, the *in vitro* model group, and the SM injection treatment group. After the cells adhered to the substrate, the in vitro model group was treated with fresh medium containing 10 ng/mL of TGF-β1 (PEPROTECH, United States), whereas the control group was given an equal amount of fresh medium without any additives. Meanwhile, the SM injection treatment group was exposed to fresh medium containing both 10 ng/mL of TGF-β1 and an appropriate concentration of the SM injection. 48 h later, the intervention was terminated, and fibroblasts from each group were harvested for further studies.

### 2.11 qRT-PCR experiment

qRT-PCR was performed to validate the key targets of SM injection in treating IPF. After the intervention, total RNA was extracted from the fibroblasts of each group using Trizol reagent (Thermo Fisher Scientific, United States). This process—including grouping, intervention, and RNA extraction—was repeated 3 times to make sure that each group had 3 separate RNA samples from different batches of HLFs. Subsequently, complementary DNA was synthesized from these RNA samples using the Evo M-MLV Reverse Transcriptase Premix Kit (Agbio, China). qRT-PCR analyses were then conducted on a qTOWER real-time PCR system utilizing the SYBR Green Premix Pro Taq HS qPCR Kit (Agbio, China), with GAPDH serving as the internal control for normalization. Additionally, the significant increase in *α*-SMA and Fn1 mRNA levels in the *in vitro* model group indicated successful modeling, given their close association with pulmonary fibrosis extent and progression. The 2^−△△Ct^ method was employed to quantify the mRNA levels of the interested genes. Primer sequences are provided in Table 1.

**Table 1:** Primer information.

Target name	Primer		Species
Fn1	F	ACAACACCGAGGTGACTGAGAC	Human
	R	GGACACAACGATGCTTCCTGAG	
α-SMA	F	CTATGCCTCTGGACGCACAACT	Human
	R	CAGATCCAGACGCATGATGGCA	
GAPDH	F	GTCTCCTCTGACTTCAACAGCG	Human
	R	ACCACCCTGTTGCTGTAGCCAA	
MMP9	F	GCCACTACTGTGCCTTTGAGTC	Human
	R	CCCTCAGAGAATCGCCAGTACT	
IL-6	F	AGACAGCCACTCACCTCTTCAG	Human
	R	TTCTGCCAGTGCCTCTTTGCTG	
TNF-α	F	CTCTTCTGCCTGCTGCACTTTG	Human
	R	ATGGGCTACAGGCTTGTCACTC	

### 2.12 WB experiment

To validate the core targets of SM injection in treating IPF at the protein expression level, HLFs were also used for WB experiments. Following the grouping and intervention previously mentioned, total protein from each group was extracted using RIPA Lysis Buffer (Solarbio, China) with protease inhibitors. The protein concentration was then determined using a BCA kit (Beyotime, China). Based on the protein concentration, the sample volume was adjusted to ensure that each group sample contained equal amounts of protein. The protein samples were next combined with 5 × loading buffer (Beyotime, China) and heated at 100°C in a water bath for denaturation. Afterwards, the samples were loaded onto 4–20% SDS-PAGE precast protein gels (ACE, China) for electrophoresis, followed by protein transfer to PVDF membranes. The membranes were blocked for 1 h using 5% skim milk and then cut according to protein sizes. They were washed 3 times with TBST before being incubated with primary antibodies overnight at 4°C. After another 3 TBST washes, the membranes were cultured with secondary antibodies for 1 h at room temperature and washed 3 additional times with TBST. Finally, the protein bands on membranes were developed with a chemiluminescence reagent (Beyotime, China) and detected with an imager (LAS 500, GE, United States). The information on antibodies is listed in Table 2. The experiments, including grouping, intervention, and WB, were repeated in triplicate using different batches of HLFs. As in the qRT-PCR experiment, GAPDH was used as the internal control, and elevated SMA and Fn1 levels indicated successful establishment of the *in vitro* fibrosis model. The protein expression levels of *α*-SMA, Fn1, MMP9, IL-6, and TNF-α were semi-quantitatively assessed and analyzed using ImageJ software (55). After measuring the integrated density and normalizing the data, the relative protein levels of the target genes for each group were calculated and then statistically compared across different groups using GraphPad Prism 8 software.

**Table 2:** Antibody information.

Name	Type	Source	Identifier	Species
GAPDH	Primary antibody	Abcam/Britain	ab181602	Rabbit
α-SMA	Primary antibody	Abcam/Britain	ab7817	Mouse
Fn1	Primary antibody	Abcam/Britain	ab45688	Rabbit
MMP9	Primary antibody	Proteintech/China	10375-2-AP	Rabbit
IL-6	Primary antibody	Proteintech/China	21865-1-AP	Rabbit
TNF-α	Primary antibody	Abcam/Britain	ab1793	Mouse
Gout anti-Rabbit IgG	Secondary antibody	Abcam/Britain	ab205718	–
Gout anti-Mouse IgG	Secondary antibody	Abcam/Britain	ab205719	–

### 2.13 ELISA experiment

To investigate whether SM injection exerts an anti-inflammatory effect in the treatment of IPF, ELISA experiments were conducted using HLFs from each group. We repeated the grouping and intervention 3 times, ensuring that each group included 3 HLF specimens from different batches. Cell supernatant from each specimen was collected for ELISA testing. Initially, after setting up wells for standards, zero controls, blanks, and samples on the Elisa plate, 50 μL of standards at different concentrations were added to the standard wells, 50 μL of sample diluent to the zero control wells, nothing to the blank wells, and 50 μL of the sample supernatants to the sample wells. Next, 100 μL of horseradish peroxidase-conjugated detection antibody was added to the standard, zero control, and sample wells (excluding the blank wells). The plate was then covered with a seal and incubated at 37°C in the dark. After 60 min, the seal was removed and the plate was washed using an automatic plate washer. Subsequently, substrates A and B (provided in assay kits) were combined in a 1:1 ratio, and 100 μL of the mixture was added to each well. The wells were covered again and incubated at 37°C in the dark for 15 min. To conclude the assay, 50 μL of stop solution was applied to all wells, and the optical density (OD) value of each well was measured with the microplate reader. The concentrations of target cytokines in each group were calculated based on their corresponding standard curves. The data were imported into GraphPad Prism 8 software for statistical analysis. Details of the ELISA kits used are provided in Table 3.

**Table 3:** ELISA kit information.

Name	Source	Identifier	Species
IL-6	Ruixin Biotech/China	RX106126H	Human
IL-1β	Ruixin Biotech/China	RX106152H	Human
IL-10	Ruixin Biotech/China	RX103064H	Human

### 2.14 Detection of reactive oxygen species

To explore whether SM injection plays an anti-oxidative role in the treatment of IPF, we carried out ROS detection in HLFs from each group. The HLFs were treated with the indicated interventions (Section 2.10) for 48 h, then washed twice with serum-free medium, stained with a 1:1000 dilution of DCFH-DA Solution (Beyotime, China), and incubated at 37°C for 20 min. Next, the cells were washed 3 additional times with serum-free medium. Observations and imaging were performed under low-light conditions using fluorescence microscopy. This experiment was also replicated 3 times with different batches of HLFs. Cellular ROS levels were semi-quantitatively assessed by measuring the mean fluorescence intensity using ImageJ software (56). Comparative analyses across different groups were performed utilizing GraphPad Prism 8 software.

### 2.15 Measurement of total glutathione (GSH) content

GSH is an important intracellular antioxidant that neutralizes ROS and maintains redox balance. Total GSH levels reflect the cellular antioxidant capacity and the extent of oxidative damage. For example, increased total GSH levels indicate improved cellular antioxidant ability and reduced oxidative stress (57). To further validate the antioxidant activity of SM injection in the process of anti-fibrosis, we measured total GSH levels in HLFs from each group using the DTNB-GSSG recycling assay (58) with a GSH Assay kit (Beyotime, China). Briefly, after interventions as described in Section 2.10, cells from each group were divided into two portions: one for protein concentration measurement using the BCA kit (Beyotime, China) and the other for total GSH measurement. The cells were centrifuged to collect the pellet, and a protein removal reagent (provided in the GSH assay kit) was added at three times the volume of the pellet. The mixture underwent two freeze–thaw cycles using liquid nitrogen and a 37°C water bath, followed by centrifugation to collect the supernatant. The supernatant was then reacted with kit reagents at room temperature for 25 min. Absorbance at 412 nm was measured, and total GSH concentration was calculated based on the absorbance value, standard curve, and formula provided in the kit instructions. Finally, the total GSH content per gram of protein in each group was used for subsequent statistical analysis.

### 2.16 Statistical analysis

The continuous data, which were presented as mean ± SEM, were analyzed using ANOVA. Categorical data were subjected to Fisher’s exact test. R v4.3.1 and GraphPad Prism 8 software were employed to conduct the data analysis. The level of statistical significance was determined to be *p* < 0.05.

## 3 Results

### 3.1 Active components and targets of SM injection

We searched the TCMSP database and screened for compounds with DL ≥ 0.18. Additionally, components such as danshensu and protocatechualdehyde were manually selected based on the literature (40, 41). A total of 138 active ingredients of SM injection were identified, which are detailed in Supplementary Table S1. The related targets for each identified compound were also collected from the TCMSP database. After removing duplicate targets, 215 unique drug targets were obtained, as listed in Supplementary Table S2.

### 3.2 Compound-target-pathway network establishment

**Figure 1:**
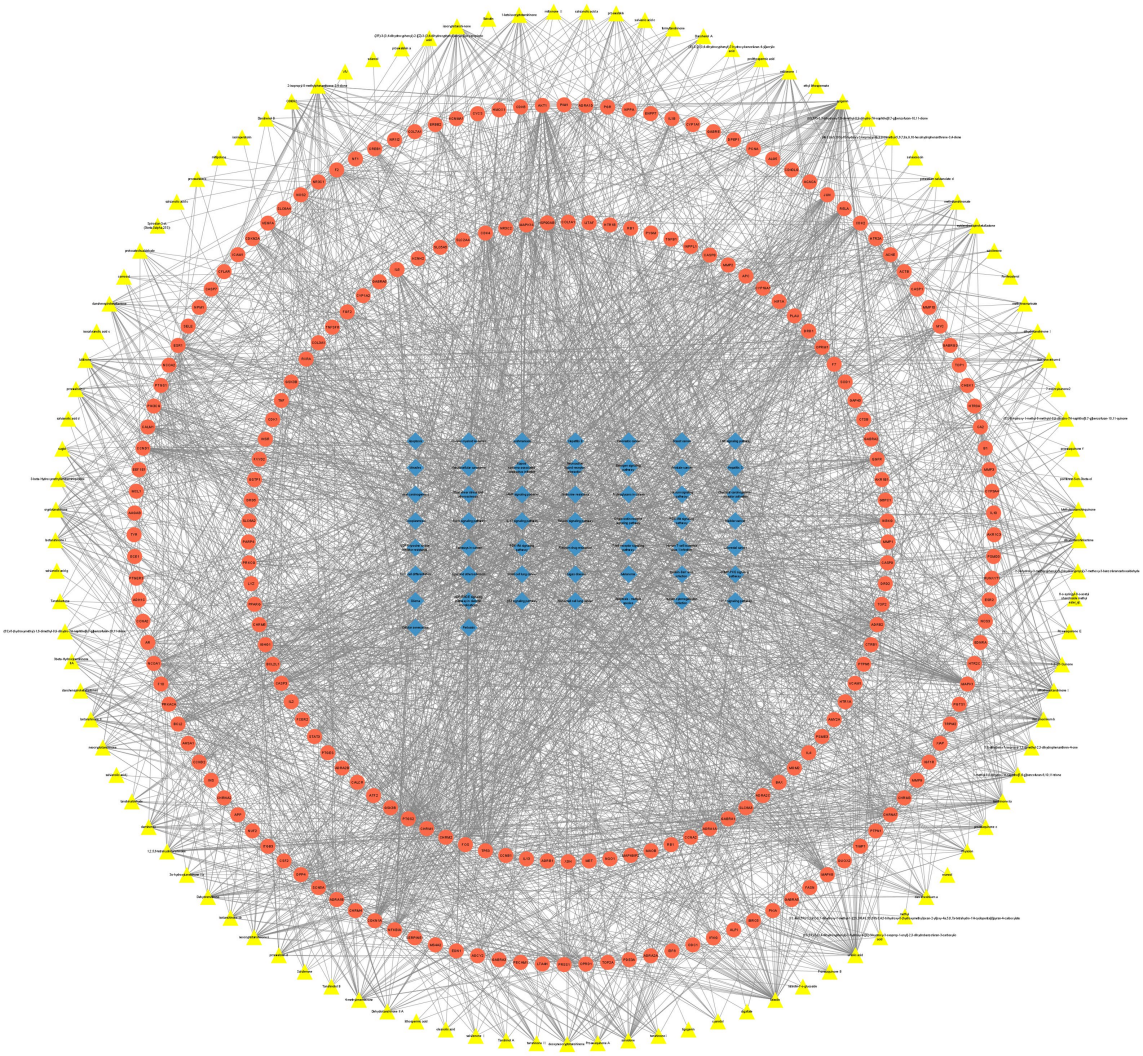
The compound-target-pathway network constructed with selected compounds, their targets, and related pathways.

Using the DAVID database, we conducted KEGG analysis to investigate pathways associated with the targets of the selected compounds. This analysis yielded the top 50 KEGG pathways associated with these targets, as detailed in Supplementary Table S3. Next, we constructed a compound-target-pathway network using Cytoscape software to visualize the interactions between the 138 active compounds in SM Injection, their 215 targets, and the top 50 KEGG pathways, as illustrated in Figure 1.

### 3.3 Selection of IPF-related genes

Using “idiopathic pulmonary fibrosis,” “familial idiopathic pulmonary fibrosis,” “exacerbation of idiopathic pulmonary fibrosis,” “chronic idiopathic pulmonary fibrosis,” “idiopathic pulmonary fibrosis (acute form),” “diffuse idiopathic pulmonary fibrosis,” and “idiopathic interstitial pneumonias” as keywords, we collected 889 IPF-related genes after removing redundant genes.

### 3.4 Acquisition of the potential targets of SM injection in treating IPF

**Figure 2:**
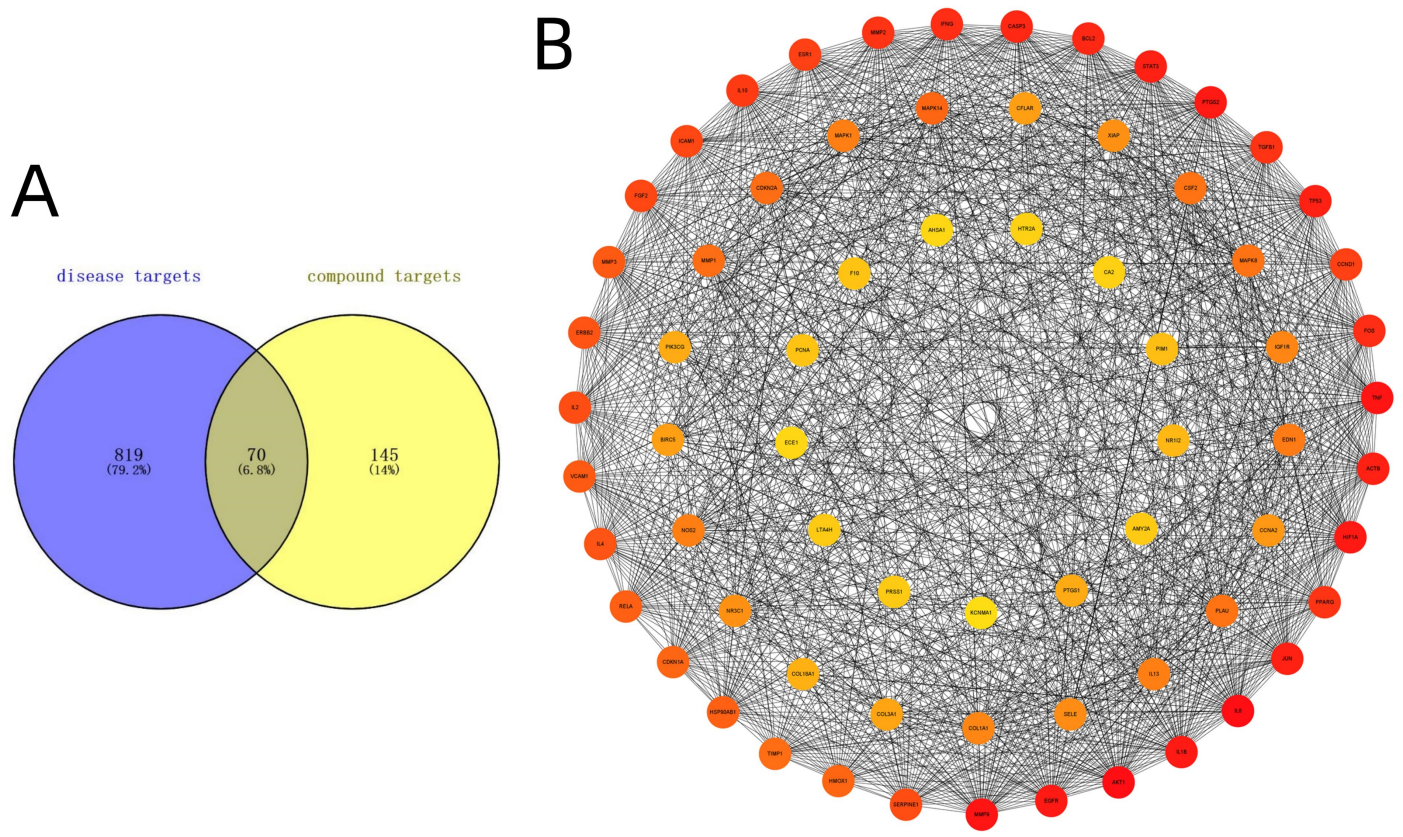
**(A)** Venn diagram intersecting 70 target genes between IPF-related genes and SM injection targets. **(B)** The PPI network constructed with 70 intersected target genes.

To explore the potential targets of SM injection in IPF treatment, we performed a Venn diagram analysis to identify the overlap between IPF-related genes and SM injection targets. The analysis identified 70 overlapping genes (see Figure 2A; Supplementary Table S4), which were regarded as potential therapeutic targets for IPF.

### 3.5 Establishment of PPI network and identification of key targets

A PPI network was constructed by Cytoscape 3.9.1 software to visualize the relationships among the intersected target genes. The PPI network dataset consisted of 69 nodes and 1,235 edges (Figure 2B). By applying the 6 algorithms of the cytoHubba plugin, we determined the top 5 candidate core targets for each method (Table 4). After intersecting the targets identified by the 6 algorithms, we finally acquired 3 common key targets: MMP9, IL-6, and TNF-*α*.

**Table 4:** Top 5 candidate key targets in cytoHubba.

Rank	Algorithms in cytoHubba					
	Degree	Radiality	Closeness	Stress	EPC	MNC
1	*IL-6*	*IL-6*	*IL-6*	*IL-6*	*TNF*-α	*IL-6*
2	*AKT1*	*AKT1*	*AKT1*	*AKT1*	*MMP9*	*AKT1*
3	*MMP9*	*MMP9*	*MMP9*	*MMP9*	*IL-6*	*MMP9*
4	*TNF*-α	*TNF*-α	*TNF*-α	*TNF*-α	*PTGS2*	*TNF*-α
5	*PTGS2*	*PTGS2*	*PTGS2*	*FOS*	*JUN*	*PTGS2*

The common key targets were marked in red.

### 3.6 GO and KEGG analyses of key therapeutic targets

**Figure 3:**
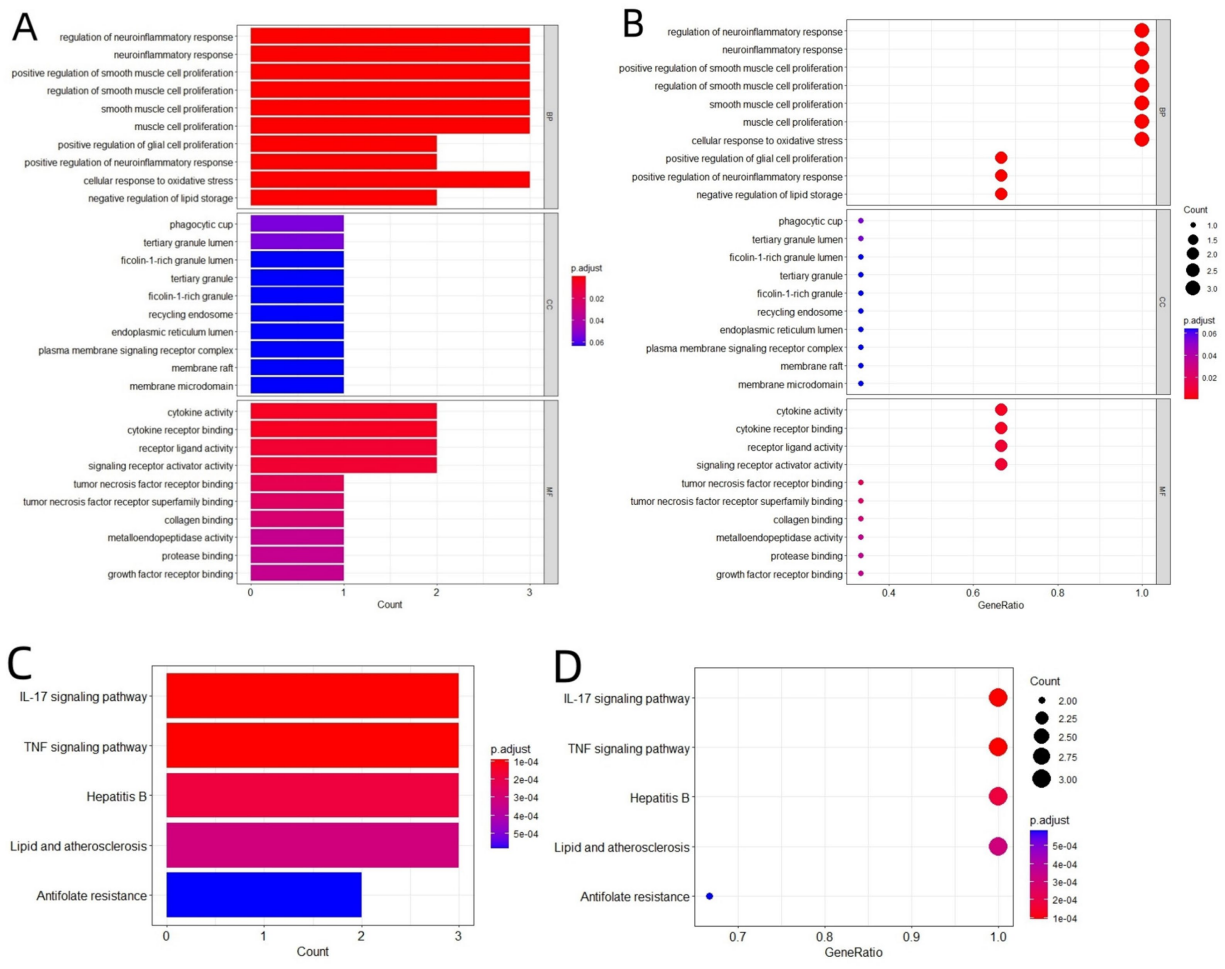
Enrichment analyses of the key targets. **(A)** Bar plot of GO analysis of the key targets. **(B)** Dot plot of GO analysis of the key targets. **(C)** Bar plot of KEGG analysis of the key targets. **(D)** Dot plot of KEGG analysis of the key targets.

We conducted GO and KEGG functional enrichment analyses on the 3 key therapeutic targets (Date: April 13th, 2024), with results shown in Figures 3A–D. The primary BP pathways involved the regulation of neuroinflammatory responses, smooth muscle cell proliferation, muscle cell proliferation, glial cell proliferation, cellular response to oxidative stress, and negative regulation of lipid storage. CC analysis revealed significant enrichment in phagocytosis-related membrane structures (phagocytic cup), specialized leukocyte organelles (tertiary granules and ficolin-1-rich granules), signaling components (recycling endosomes, membrane rafts, membrane microdomains, and plasma membrane signaling receptor complex), and endoplasmic reticulum structures. MF analysis highlighted enrichment in activities related to cytokines, growth factors, signal transduction, collagen binding, protease binding, and metalloproteinases. KEGG analysis further demonstrated significant enrichment of key targets in pathways associated with hepatitis B, antifolate resistance, lipid and atherosclerosis, as well as IL-17 and TNF-*α* signaling.

### 3.7 Molecular docking results

Using the compound-target-pathway network established in Section 3.2, the degree value of each compound was calculated using Cytoscape software. The top 15 compounds with the highest degree scores were selected as key active components, as demonstrated in Supplementary Table S5. It’s worthy to note that since 1,2,5,6-tetrahydrotanshinone, dan-shexinkum D, and neocryptotanshinone ii have identical degree values, a total of 16 components were ultimately screened. To identify potential active ingredients in SM injection for IPF treatment, we conducted molecular docking on the 16 components, using the 3 core therapeutic targets (MMP9, IL-6, and TNF-*α*) as receptors. The results, presented in Table 5, revealed a total of 48 docking pairs exhibiting good binding affinities (energy ≤ − 5.0 kcal/mol) between the components and the target proteins. This suggests that all 16 components have high binding affinities with the 3 key targets.

**Table 5:** Docking results for the important components of SM and key targets.

Component	MMP9	IL-6	TNF-α
	Binding energy (kcal/mol)		
Apigenin	−10.4	−6.8	−7
Luteolin	−10.6	−7.2	−6.9
Ursolic acid	−7.8	−7.9	−7.4
Dihydroisotanshinonei	−8.6	−7.2	−7.3
Tanshinone ii A	−9.3	−7.6	−8.2
Dan-shexinkum B	−10.1	−7.1	−6.9
Salviolone	−8.7	−7.5	−7.6
Dihydrotanshinlactone	−9.3	−7.4	−6.8
2-isopropyl-8-methylphenanthrene-3,4-dione	−8.5	−7.3	−7.2
4-methylenemiltirone	−6.6	−6.2	−6.4
1,2-DT-Quinone	−9.2	−7.3	−7.9
Isocryptotanshi-none	−8.5	−7.3	−7.2
Cryptotanshinone	−8.2	−7.3	−7.8
1,2,5,6-tetrahydrotanshinone	−8.8	−7.4	−7
Dan-shexinkum D	−8.2	−8.1	−6.9
Neocryptotanshinone ii	−7.6	−7.1	−7.2

**Figure 4:**
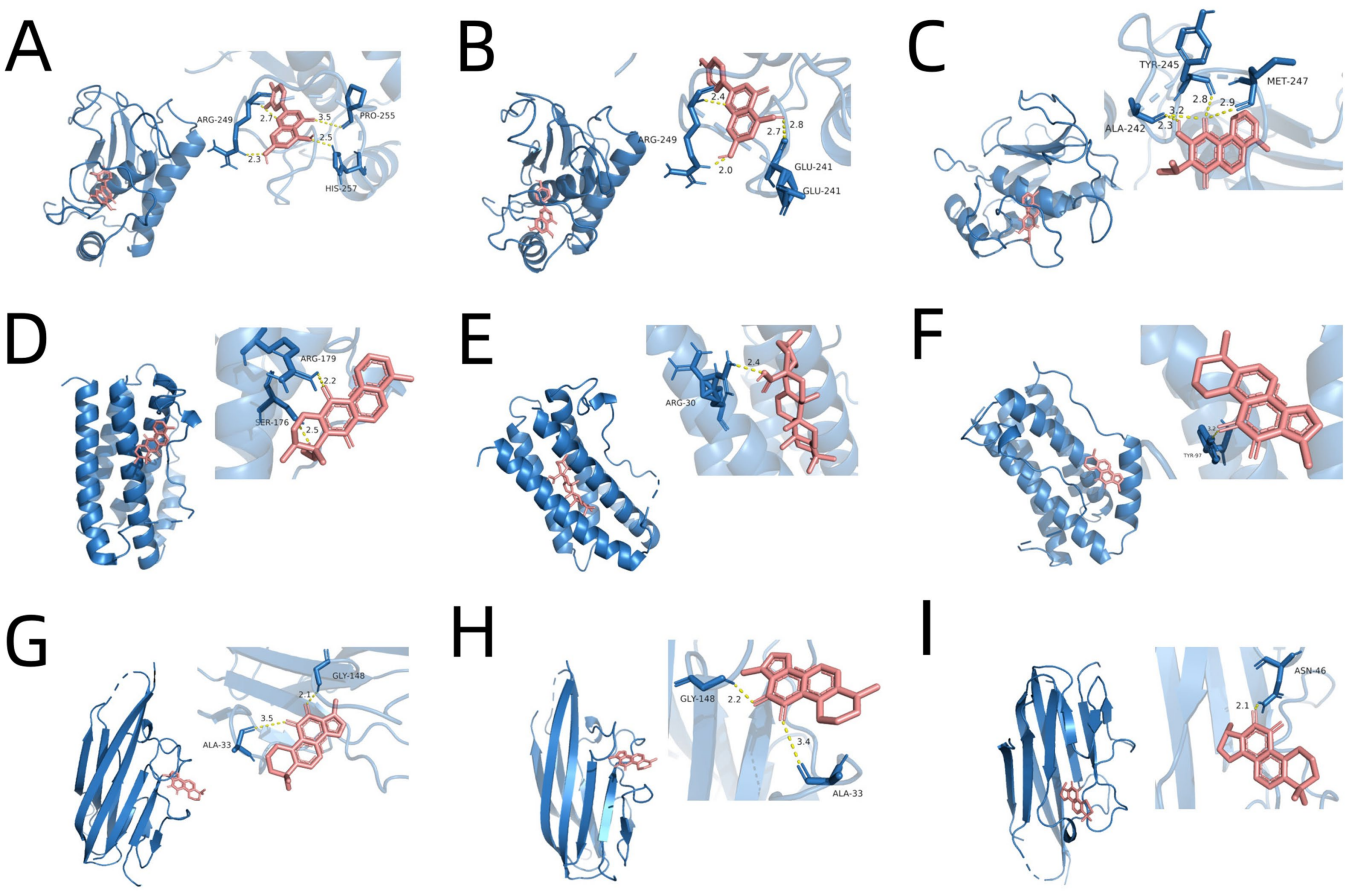
Molecular docking between key targets and important components of SM. **(A)** Molecular docking schematic for luteolin with MMP9. **(B)** Molecular docking schematic for apigenin with MMP9. **(C)** Molecular docking schematic for dan-shexinkum B with MMP9. **(D)** Molecular docking schematic for dan-shexinkum D with IL-6. **(E)** Molecular docking schematic for ursolic acid with IL-6. **(F)** Molecular docking schematic for tanshinone IIA with IL-6. **(G)** Molecular docking schematic for tanshinone IIA with TNF-α. **(H)** Molecular docking schematic for 1, 2-DT-quinone with TNF-α. **(I)** Molecular docking schematic for cryptotanshinone with TNF-α.

Notably, 38 pairs showed strong binding abilities (energy ≤ − 7.0 kcal/mol). The top 3 docked complexes with the strongest binding affinities for each target are visualized in Figure 4. Among these compounds, luteolin, apigenin, and dan-shexinkum B exhibited the highest binding affinities with MMP9 (Figures 4A–C), whereas dan-shexinkum D, ursolic acid, and tanshinone IIA were identified as the strongest binders to IL-6 (Figures 4D–F). As for TNF-*α*, tanshinone IIA, 1,2-DT-quinone, and cryptotanshinone demonstrated the most potent binding interactions (Figures 4G–I).

### 3.8 Results of CCK-8 assay

**Figure 5:**
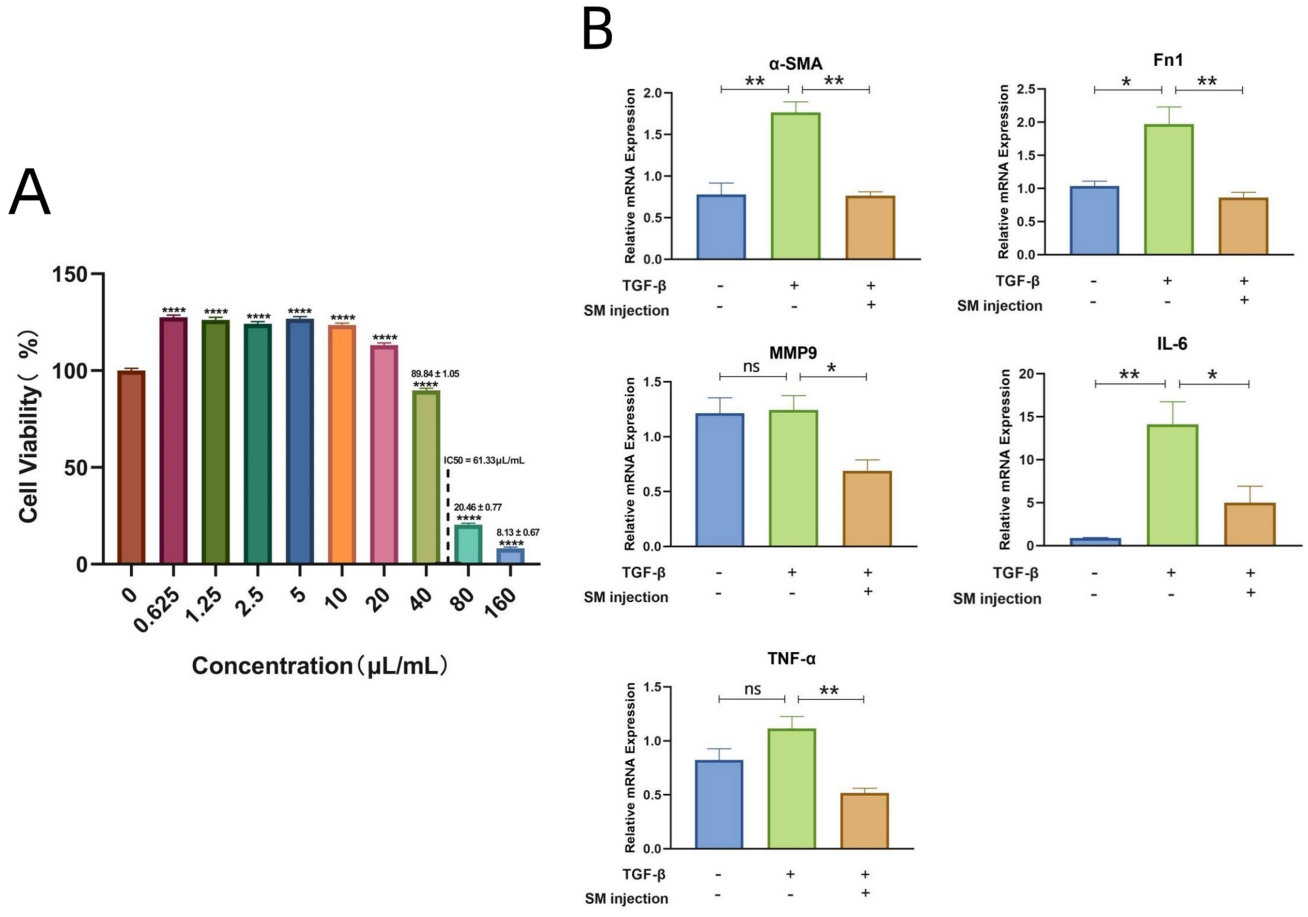
**(A)** Bar plot visualizing the cell viability data of HLFs across various concentrations of SM injection. **(B)** Bar plots depicting mRNA levels of α-SMA, Fn1, MMP9, IL-6, and TNF-α across different groups. * *p* < 0.05, ** *p* < 0.01, **** *p* < 0.0001, and ns, no significance.

The CCK-8 assay results demonstrated that SM injection exhibited a dual effect on HLF cells: low doses promoted cell proliferation, while higher concentrations induced cytotoxicity. Specifically, at concentrations above 40 μL/mL, a reduction in cell viability was observed, indicating the onset of cytotoxic effects (Figure 5A). When the concentration reached 160 μL/mL, cell viability dropped to less than 10% compared to the control group (Figure 5A). At higher concentrations, SM injection showed dose-dependent cytotoxicity, and the IC50 value of SM injection in HLF cells was calculated as 61.33 μL/mL. Based on these findings, a concentration of 40 μL/mL, which did not exhibit significant cytotoxicity while potentially maintaining biological activity, was selected for further study.

### 3.9 Results of qRT-PCR

To validate the reliability of the key targets of SM injection in treating IPF, we examined the relative mRNA levels of these targets in each group using qRT-PCR. The results demonstrated a significant rise in the mRNA levels of Fn1 and *α*-SMA in the TGF-β1-stimulated group compared to the control group, indicating the successful establishment of an *in vitro* model of lung fibrosis. Meanwhile, SM injection exhibited a potential anti-fibrotic effect on TGF-β1-stimulated fibroblasts, as evidenced by the marked decrease in the mRNA expression of *α*-SMA and Fn1 in the treatment group compared to the model group (Figure 5B). Furthermore, the relative mRNA levels of MMP9, IL-6, and TNF-α were downregulated in the treatment group compared to the model group (Figure 5B).

### 3.10 Results of WB

**Figure 6:**
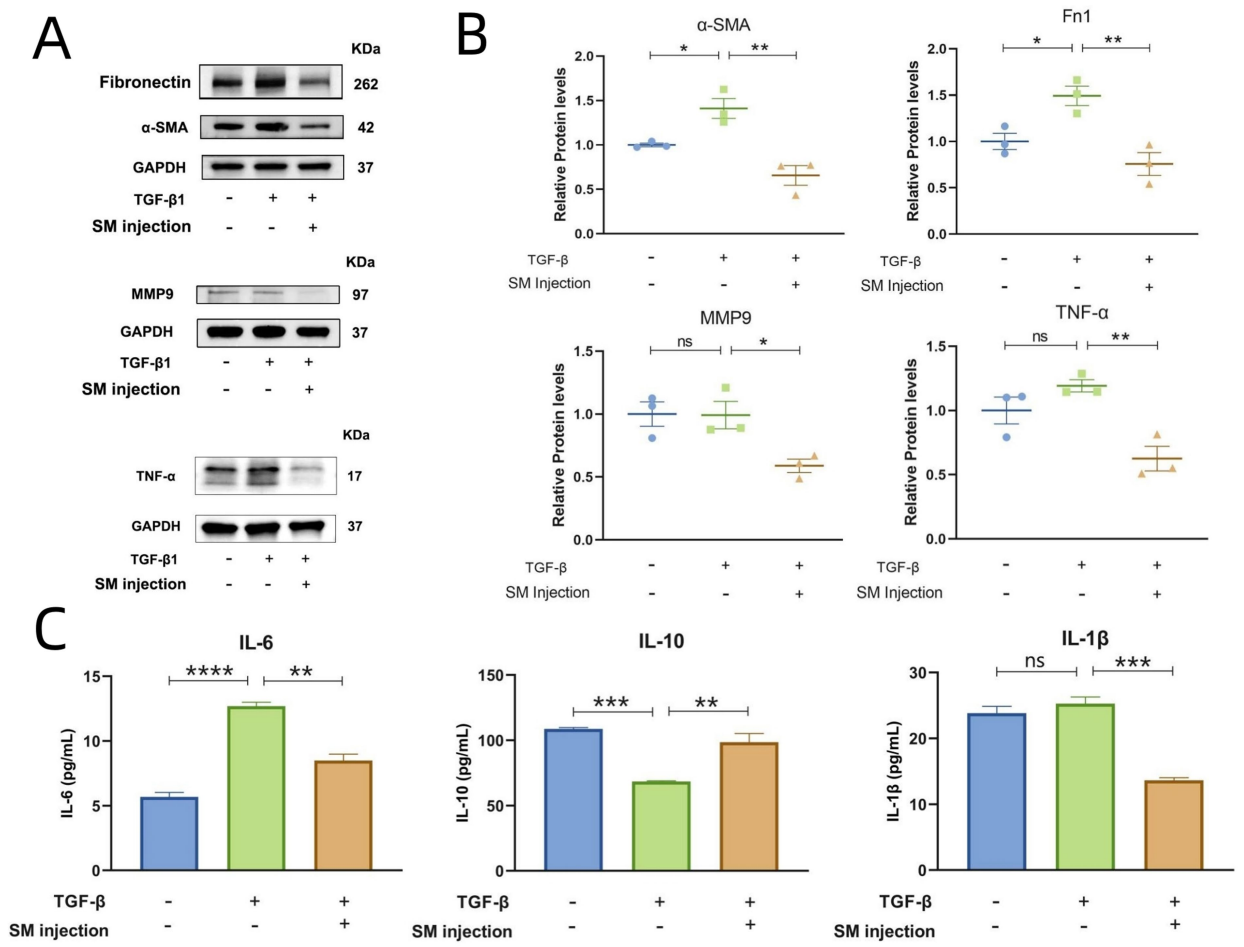
Results of WB and ELISA. **(A)** Bands for Fn1, α-SMA, MMP9, and TNF-α across groups. **(B)** Scatter plots presenting the relative protein levels of α-SMA, Fn1, MMP9, and TNF-α across groups. **(C)** Bar graphs of IL-6, IL-10, and IL-1β concentrations across groups. * *p* < 0.05, ** *p* < 0.01, *** *p* < 0.001,**** *p* < 0.0001, and ns, no significance.

We further conducted WB experiments to compare the protein expression levels of key targets across the groups. In the WB analysis, α-SMA and Fn1 were detected at 42 kDa and 262 kDa, respectively, in all 3 groups. The band intensity image and scatter plots revealed that the protein expression levels of α-SMA and Fn1 were highest in the model group and lowest in the treatment group (Figures 6A,B). These findings support the successful establishment of a pulmonary fibrosis model and demonstrate the anti-fibrotic efficacy of SM injection at the protein level. For the key targets, MMP9 and TNF-α were detected at molecular weights of 97 kDa and 17 kDa, respectively. The band intensities and statistical analyses suggested a downregulation in the protein expression levels of MMP9 and TNF-α in the treatment group compared to the model group (Figures 6A,B). However, no bands for IL-6 were detected in any of the groups.

### 3.11 Results of ELISA

Given that IL-6 is a secretory protein and is typically less expressed intracellularly, we performed ELISA experiments to measure the levels of IL-6 in the supernatants of HLFs from each group. Additionally, to explore whether SM injection plays an anti-inflammatory role in the treatment of IPF, we assessed the concentrations of IL-10 and IL-1β in the same supernatants. The results revealed a considerable reduction in the levels of the pro-inflammatory cytokines IL-6 and IL-1β in the treatment group in comparison to the model group, while the concentrations of the anti-inflammatory cytokine IL-10 notably increased in the treatment group (Figure 6C).

### 3.12 Results of ROS detection and total GSH measurement

**Figure 7:**
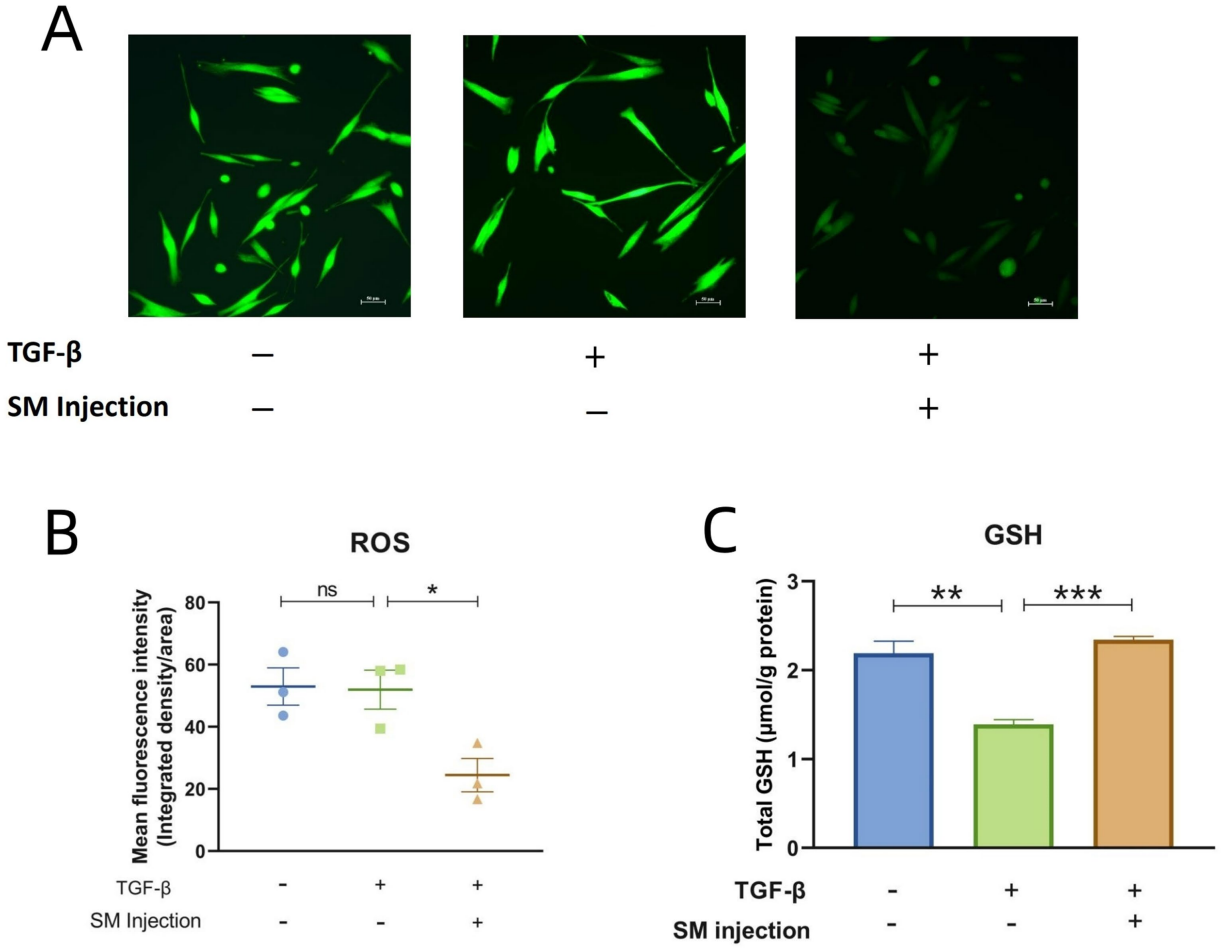
Results of ROS detection and total GSH measurement. **(A)** Fluorescence microscopy images of 3 groups. **(B)** Scatter plot presenting the mean fluorescence intensity across 3 groups. **(C)** Bar graphs of total GSH content across 3 groups. * *p* < 0.05, ** *p* < 0.01, *** *p* < 0.001, and ns, no significance.

In fluorescence microscopy, ROS activity in cells is visualized by green fluorescence. The images represent the relative ROS levels in fibroblasts across different experimental groups, with fluorescence intensity serving as an indicator of ROS activity. Specifically, increased fluorescence intensity correlates with higher ROS levels and suggests elevated oxidative stress, while decreased intensity reflects lower ROS levels. As illustrated in Figure 7A, the treatment group demonstrates a marked reduction in ROS levels in comparison to the model group. This observation is further supported by the ImageJ and statistical analysis presented in Figure 7B.

Regarding the levels of total GSH, the model group exhibited a significant decrease compared to the control group. In contrast, the treatment group with SM injection showed a notable increase in total GSH content compared to the model group (Figure 7C).

## 4 Discussion

Due to the limited effectiveness of current treatments for IPF and its increasing incidence, IPF poses a public health challenge and remains “incurable.” Consequently, it is imperative to seek new therapeutic medications that effectively inhibit the progression of IPF. Given that inflammation, oxidative stress, and immunity are important contributors to IPF, and SM is noted for its anti-inflammatory and antioxidant effects, investigating the potential benefits and mechanisms of SM injection for IPF may be advantageous for practical use and clinical treatment.

Using network pharmacology analysis, we identified 138 active components in SM injection and determined 215 potential targets for these components. The DAVID database was used to conduct KEGG pathway analysis on these targets, leading to the identification of the top 50 enriched pathways. The KEGG analysis demonstrates that these targets participate in diverse pathways, including those associated with immune response, inflammation, signal transduction, metabolism, cellular behavior, cancer, and infection. This suggests that SM injection could exert therapeutic effects in various diseases through multiple targets. When these targets were intersected with IPF-related disease genes via Venn analysis, 70 potential therapeutic targets for IPF were obtained. This result, along with studies on individual components of SM, supports the potential therapeutic value of SM injection for IPF (20–31, 59). By acting on these specific targets, SM injection may impact different stages of IPF, thus becoming a novel multi-targeted therapeutic medicine for IPF.

To find the most important target genes among the 70 therapeutic targets, we employed Cytoscape to construct a PPI network for these targets. From this network, we determined the key targets using 6 different algorithms. By taking the intersection of the top 5 genes identified by each algorithm, we ultimately screened IL-6, TNF-*α*, and MMP9 as the key therapeutic targets.

IL-6 (interleukin-6) is a cytokine involved in diverse biological processes, including immune regulation, inflammation, and hematopoiesis. It is also recognized as a key factor in the progression of lung fibrosis, with elevated levels observed in IPF patients and mouse models of lung fibrosis (60–62). In the pathological process of IPF, IL-6 drives disease progression through multiple mechanisms. Firstly, it exerts pro-fibrotic effects through the classic JAK/STAT pathway, which mediates inflammatory responses, fibroblast activation, EMT, and oxidative stress (62–64). Secondly, IL-6 regulates cellular functions through the PI3K/AKT and MAPK pathways (63, 64). The PI3K/AKT pathway promotes fibrosis by enhancing fibroblast survival, inhibiting apoptosis, and modulating ECM metabolism (65), while the MAPK pathway amplifies pro-inflammatory signals and induces epithelial cell senescence, further exacerbating fibrotic progression (66, 67). IL-6 also synergizes with TGF-*β* to enhance fibrotic responses by activating the Smad pathway (62). Additionally, IL-6 participates in abnormal tissue regeneration and remodeling by modulating SFKs, YAP, and Notch pathways and promoting macrophage polarization toward the M2 phenotype (64, 68–70). These mechanisms potentially contribute to the pro-fibrotic microenvironment.

TNF-*α* (Tumor Necrosis Factor-alpha) is another pro-inflammatory cytokine mainly synthesized by activated macrophages, and it regulates immunological responses and inflammation. Similar to IL-6, TNF-α contributes to lung fibrosis by promoting inflammation, oxidative stress, tissue remodeling, and fibroblast activation, and its levels are elevated in IPF patients and experimental models (60, 61, 71–73). However, what distinguishes TNF-α is its unique pro-fibrotic mechanisms. While TNF-α can activate the MAPK pathway (68), it primarily signals through the NF-κB pathway, which promotes the release of inflammatory and fibrotic mediators, leading to progressive fibrotic remodeling and lung tissue dysfunction (68, 74). In addition, although both TNF-α and IL-6 influence macrophage polarization, TNF-α tends to promote M1 macrophage polarization, which sustains inflammation (70, 75), whereas IL-6 drives M2 macrophage polarization. TNF-α can also upregulate TGF-*β* expression and enhance its downstream effects (76), but it does not directly activate the Smad pathway like IL-6 does. Furthermore, TNF-α exhibits a dual role in apoptosis: it promotes lung fibroblast survival while inducing epithelial cell death (77, 78), highlighting its uniqueness in fibrosis pathogenesis compared to IL-6.

MMP9 (Matrix Metallopeptidase 9) is an enzyme involved in the breakdown of ECM in various physiological processes such as wound healing, tissue remodeling, and immune response modulation (79). Theoretically, elevated MMP9 expression could inhibit ECM accumulation by degrading collagen and other matrix components, potentially reducing fibrosis progression. However, enhanced MMP9 activity is more commonly observed in the pathological state of pulmonary fibrosis, as elevated MMP9 expression levels have been detected in both bleomycin-induced mouse models and IPF patients (79–82). This is because MMP9 plays a multifaceted role in the pathogenesis of IPF by contributing to ECM deposition, EMT, and inflammation. Although MMP9 initially aids ECM degradation, its overexpression in IPF promotes pathological ECM deposition by activating TGF-β signaling pathway and disrupting ECM degradation-synthesis balance (83). On the other hand, MMP9 degrades basement membrane components, facilitating epithelial cell transmigration and inducing EMT (84). MMP9 is also regulated by oxidative stress and inflammation pathways such as NF-κB and MAPK, which sustain a chronic inflammatory state and promote fibrosis progression (8, 85–89).

GO and KEGG analyses reveal that the 3 key therapeutic targets are mainly involved in activities and pathways related to cytokines, growth factors, and signal transduction; regulation of immune and inflammatory responses; cellular responses to oxidative stress; myofibroblast proliferation; as well as collagen synthesis and deposition. All of these are strongly connected with fibrosis development (4). This suggests that the MMP9, IL-6, and TNF-*α* collectively exert multiple effects in driving lung fibrosis progression. More importantly, the interactions and synergistic effects among these molecules may create a self-reinforcing cycle that worsens fibrosis. For example, pro-inflammatory cytokine TNF-*α* induces MMP9 overexpression, which in turn amplifies inflammation (85, 87–89). IL-6 and TNF-α interact and synergistically promote inflammation, oxidative stress, and fibroblast activation (7, 8). Together, these interactions and enriched pathways form a complex signaling network that sustains and facilitates fibrotic processes. It indicates that therapeutic strategies targeting the bioactivities of these key targets or their associated pathways may have potential as new treatments for IPF.

Based on the results of qRT-PCR and WB, the expression levels of α-SMA and Fn1 increased in the model group and decreased in the SM injection treatment group, confirming the anti-fibrotic effects of SM injection. A downregulation of MMP9, IL-6, and TNF-α was also observed in the treatment group compared to the model group, validating that SM injection could alleviate fibrosis by modulating these targets. However, how SM injection acts on these targets to exert its anti-fibrotic effects may involve complicated mechanisms. First of all, unlike antibody-based inhibitors that directly bind to target proteins with high specificity, molecular docking analysis reveals that the active small molecules in SM injection may bind to the 3 key targets with comparatively lower specificity (Table 5; Figure 4). These bindings may alter the conformation of the target proteins, inhibiting their activity or suppressing related signaling pathways (51, 90, 91). This hypothesis is supported by indirect evidence showing that some active small molecules in SM have been shown to prevent fibrosis progression through the inhibition of signaling pathways involving IL-6, TNF-*α*, and MMP9. For instance, tanshinone IIA alleviates pulmonary fibrosis by modulating TGF-*β*/Smad, JAK/STAT, NF-κB, and MAPK signaling pathways (20–24). Cryptotanshinone mitigates lung fibrosis by regulating TGF-β/Smad and JAK/STAT signaling pathways (31). Danshensu suppresses fibrosis via MAPK pathway (25), while salvianolic acid B exerts its anti-fibrotic effects through NF-κB pathway (59). As previously discussed, these pro-fibrotic and pro-inflammatory pathways are associated with the modulation of the key targets. Therefore, SM injection may exert its anti-fibrotic effects by influencing the key targets and their related pathways, likely through its active components. Compared to monoclonal antibodies, the small molecule compounds offer several advantages, including oral administrability, better tissue penetration, cost efficiency, modifiability, and multi-target effects (92). However, experimental validation is needed to confirm these binding interactions and the precise therapeutic mechanisms of small molecules, which could involve structural biology studies and functional assays.

In addition, the anti-inflammatory and antioxidant activities of SM injection were observed during its anti-fibrosis process. Supporting this, ELISA results indicated that SM injection significantly reduced the pro-inflammatory cytokine IL-1β and increased the anti-inflammatory cytokine IL-10 compared to the model group. It also demonstrated antioxidative properties by lowering ROS levels and elevating total GSH levels. These antioxidant and anti-inflammatory effects of SM injection probably involve the modulation of MMP9, IL-6, and TNF-α, as the 3 targets not only participate in inflammation pathways previously mentioned but also interact with oxidative stress (85, 93, 94). Such activities of SM injection are likely to exert a protective effect on fibroblasts, creating a microenvironment that mitigates fibrosis.

There are several limitations to this research. Firstly, while network pharmacology provides a valuable framework for understanding the complex mechanisms of drug action, it is important to note its potential biases and inherent limitations, such as database incompleteness, false-positive interactions, context-specificity of interactions, and overreliance on computational predictions (95). Since our conclusions are based on network pharmacology analyses, further experiments—such as target modulation assays and multi-omics data integration—are needed to confirm whether SM injection exerts anti-fibrotic functions primarily through these key targets. Secondly, the molecular docking results are computer-simulated and remain hypothetical. For now, neither our research nor existing studies on the SM components for treating fibrosis have included functional assays or structural biology studies, leaving the reliability of the in silico findings unsupported by empirical evidence. Thirdly, whether the observed antioxidant and anti-inflammatory effects of SM injection contribute to the alleviation of pulmonary fibrosis, or are merely associated phenomena during the treatment, requires experimental validation. This could include pathway inhibition, time-course analysis, and reference drug comparison. Additionally, despite the observed anti-fibrotic effects of SM injection in the fibroblast model of pulmonary fibrosis, its *in vivo* efficacy remains uncertain. Thus, in vivo experiments are necessary to provide essential data for further application in IPF.

Last but not least, although many drugs with anti-inflammatory, antioxidant, and immunomodulatory properties have shown promising anti-fibrotic effects in cell and animal studies, most of these drugs have not met primary endpoints in clinical trials and have sometimes caused adverse effects (96–98). This discrepancy may result from the fact that most cell and animal experiments typically use TGF-*β* and bleomycin to induce fibrosis through inflammatory stimulation, which does not fully replicate the pathological state of IPF (7). Regardless of these concerns, several drugs targeting inflammatory and immune processes are currently undergoing clinical trials, with some showing therapeutic outcomes (99–101). Therefore, high-quality randomized controlled trials are indispensable in the future to confirm the clinical benefits of SM injection in IPF patients.

Nevertheless, compared to previous studies focusing on individual active components of SM, this work advances the field by exploring the synergistic effects of SM as a whole, which may exert a stronger therapeutic impact on IPF. Utilizing bioinformatics and network pharmacology, this study uncovers the multi-target and multi-pathway mechanisms of SM injection in treating pulmonary fibrosis, providing a more comprehensive understanding of its therapeutic potential. This research not only provides insights into the development of innovative TCM-based therapies but also bridges traditional medicine and modern scientific research, offering a promising strategy for IPF management.

## 5 Conclusion

This research identified and validated MMP9, IL-6, and TNF-*α* as key therapeutic targets for SM injection in treating IPF through network pharmacology analyses and experiments. Enrichment analyses show that these 3 targets primarily contribute to inflammation, oxidative stress, fibroblast activation, and ECM remodeling, which play essential roles in the pathogenesis of IPF. Molecular docking reveals that the main active ingredients of SM injection exhibit high binding affinities to the key targets, suggesting their potential therapeutic effects on IPF by acting on these targets. Moreover, qRT-PCR, WB, and ELISA experiments validate that SM injection could alleviate pulmonary fibrosis by modulating the 3 targets. These findings demonstrate that SM injection may effectively reduce pulmonary fibrosis through multiple components, targets, and mechanisms, providing new TCM-based strategies for the treatment of IPF.

## Data Availability

The original contributions presented in the study are included in the article/Supplementary material, further inquiries can be directed to the corresponding authors.

## Footnotes

^1^ https://www.uniprot.org/

^2^ https://www.disgenet.org/search

^3^ https://www.rcsb.org/

^4^ https://pymol.org/

^5^ http://vina.scripps.edu/

^6^ https://pubchem.ncbi.nlm.nih.gov/

## Glossary

IPF: idiopathic pulmonary fibrosis
SM: *Salvia Miltiorrhiza*
TCMSP: Traditional Chinese Medicine Systems Pharmacology
PPI: protein–protein interaction
DAVID: Database for Annotation, Visualization, and Integrated Discovery
TCM: traditional Chinese medicine
STRING: Search Tool for the Retrieval of Interacting Genes
GO: Gene Ontology
KEGG: Kyoto Encyclopedia of Genes and Genomes
WB: Western-Blot
ECM: extracellular matrix
ROS: reactive oxygen species
EMT: epithelial-mesenchymal transition
HL: drug half-life
OB: oral bioavailability
DL: drug-likeness
BBB: blood–brain barrier
EPC: edge percolated component
MNC: maximal neighborhood component
CC: cellular component
BP: biological process
MF: molecular function
HLF: human lung fibroblast
CCK-8: cell counting kit-8
IC50: Half Maximal Inhibitory Concentration
qRT-PCR: quantitative real time polymerase chain reaction
BCA: Bicinchoninic Acid
RIPA: Radio-Immunoprecipitation Assay
TBST: tris-buffered saline with tween
OD: optical density
GSH: glutathione
DTNB-GSSG: 5,5’-Dithiobis (2-nitrobenzoic acid)-oxidized glutathione
ANOVA: Analysis of Variance
SEM: standard error of the mean
BALF: bronchoalveolar lavage fluid
