# Supplementary material for: Network pharmacology-based approach to research the effects and mechanisms of Salvia Miltiorrhiza injection against idiopathic pulmonary fibrosis
Source: Front Med (Lausanne). 2025 Jun 24;12:1569590. doi: 10.3389/fmed.2025.1569590 (PMC12235086; doi:10.3389/fmed.2025.1569590)
Supplement: Supplementary file 1 [file Table_1.docx]

**Supplementary Materials**

Table S1 the active compounds of SM Injection

| **Molecule Name** |
| --- |
| *(2R)-3-(3,4-dihydroxyphenyl)-2-[(Z)-3-(3,4-dihydroxyphenyl)acryloyl]oxy-propionic acid; (2S,3S)-2-(3,4-dihydroxyphenyl)-7-hydroxy-4-[(E)-3-hydroxy-3-oxoprop-1-enyl]-2,3-dihydrobenzofuran-3-carboxylic acid; (4bS,8aS,10S)-10-hydroxy-2-isopropyl-4b,8,8-trimethyl-5,6,7,8a,9,10-hexahydrophenanthrene-3,4-dione; (6S,7R)-6,7-dihydroxy-1,6-dimethyl-8,9-dihydro-7H-naphtho[8,7-g]benzofuran-10,11-dione; (6S)-6-(hydroxymethyl)-1,6-dimethyl-8,9-dihydro-7H-naphtho[8,7-g]benzofuran-10,11-dione; (6S)-6-hydroxy-1-methyl-6-methylol-8,9-dihydro-7H-naphtho[8,7-g]benzofuran-10,11-quinone; (E)-3-[2-(3,4-dihydroxyphenyl)-7-hydroxy-benzofuran-4-yl]acrylic acid; (Z)-3-[2-[(E)-2-(3,4-dihydroxyphenyl)vinyl]-3,4-dihydroxy-phenyl]acrylic acid; 1-ketoisocryptotanshinone; 1-methyl-8,9-dihydro-7H-naphtho[5,6-g]benzofuran-6,10,11-trione; 1,2-DT-Quinone; 1,2,5,6-tetrahydrotanshinone; 1,5-Dihydroxy-3-methylanthraquinone; 2-(4-hydroxy-3-methoxyphenyl)-5-(3-hydroxypropyl)-7-methoxy-3-benzofurancarboxaldehyde; 2-isopropyl-8-methylphenanthrene-3,4-dione; 3-beta-Hydroxymethyllenetanshiquinone; 3-epicorosolic,acid; 3beta-Hydroxytanshinone IIA; 3α-hydroxytanshinoneⅡa; 4-methylenemiltirone; 5,6-dihydroxy-7-isopropyl-1,1-dimethyl-2,3-dihydrophenanthren-4-one; 6-o-syringyl-8-o-acetyl shanzhiside methyl ester; 6-o-syringyl-8-o-acetyl shanzhiside methyl ester_qt; 7-oxoroyleanone2; 9-methyl lithospermate b; Aethiopinone; Apigenin; Baicalin; C09092; Carnosol; Cryptotanshinone; Cyanidol; Dan-shexinkum a; Dan-shexinkum b; Dan-shexinkum c; Dan-shexinkum d; Danshenol A; Danshenol B; Danshenspiroketallactone; Danshenspiroketallactoneii; Daucosterol; Dehydromiltirone; Dehydrotanshinone II A; Dehydrouvaol; Deoxyneocryptotanshinone; Digallate; DihydroisotanshinoneⅠ; Dihydrotanshinlactone; DihydrotanshinoneⅠ; Dimetbyl lithosper-mate b; Dimethyllithospermate; Epidanshenspiroketallactone; Ethyl lithospermate; Formyltanshinone; Glucosol; Henicosyl formate; HEPTACOSANE; Heriguard; Isocryptotanshi-none; Isoimperatorin; Isosalvianolic acid c; Isotanshinone I; Isotanshinone II; Isotanshinone iib; lithospermic acid; Lithospermic acid B; Luteolin; Luteolin-7-o-glucoside; Manool; Methyl(1S,4aS,5R,7S,7aS)-5,7-dihydroxy-7-methyl-1-[(2S,3R,4S,5S,6R)-3,4,5-trihydroxy-6-(hydroxymethyl)oxan-2-yl]oxy-4a,5,6,7a-tetrahydro-1H-cyclopenta[d]pyran-4-carboxylate; Methylenetanshinquinone; Methylrosmarinate; Methyltanshinonate; Microstegiol; Miltionone Ⅰ; Miltionone Ⅱ; Miltipolone; Miltirone; Miltirone Ⅱ; Monomethyl lithospermate; Neo-przewaquinone a; Neocryptotanshinone; Neocryptotanshinone ii; NSC 122421; NSC733507; Oleanolic acid; Oleanolic acid deriv; Paramiltioic acid; PENTACOSANE; Physcion; Poriferast-5-en-3beta-ol; Poriferasterol; Potassium salvianolate d; Prolithospermic acid; Przewalskin; Przewalskin a; Przewalskin b; Przewalskin c; Przewalskin d; Przewaquinone A; Przewaquinone B; Przewaquinone c; Przewaquinone E; Przewaquinone f; Saloilenone; Salviacoccin; Salvianic acid c; Salvianolic acid a; Salvianolic acid b; Salvianolic acid c; Salvianolic acid d; Salvianolic acid e; Salvianolic acid g; Salvianolic acid j; Salvianolic acid n; Salvilenone; Salvilenone Ⅰ; Salviol; Salviolone; Salvipisone; Saprothoquinone; Sclareol; Spirostan-3-ol, (3beta,5alpha,25S)-; Sugiol; Tanshilactone; Tanshinaldehyde; Tanshindiol B; Tanshinol A; Tanshinone I; Tanshinone iia; Tanshinone Ⅵ; Tigogenin; Ursolic acid; Uvaol; VIV; α-amyrin; Protocatechualdehyde; Danshensu.* |

Table S2 the targets of selected compounds in SM Injection

| **Target Gene Symbol** |
| --- |
| *F2; ESR1; AR; PPARG; PTGS2; DPP4; PRSS1; CCNA2; F7; PTPN1; PYGM; HSP90AB1; ACHE; PGR; NR3C1; NCOA2; NCOA1; CHRM1; SCN5A; OPRD1; ADRA1A; ADRB2; OPRM1; CHRNA7; CA2; NOS2; PTGS1; F10; CHRM5; CHRM2; ADRA1B; TOP2A; GABRA1; DRD1; CHRM3; NOS3; HTR3A; RXRA; HTR2A; ADRA1D; SLC6A4; PIK3CG; PRKACA; IGHG1; CHRM4; PDE3A; SLC6A3; TOP2; ESR2; MAPK14; GSK3B; CDK2; PIM1; CALM1; ADRA2A; ADRA2C; DRD2; NR3C2; RELA; AKT1; VEGFA; CCND1; BCL2; BCL2L1; FOS; CDKN1A; EIF6; BAX; CASP9; PLAU; MMP9; RB1; CDK4; TNF; JUN; CDK6; CDKN2A; EEF1E1; AHSA1; CASP3; TP53; NFKBIA; ODC1; MDM2; MMP1; HIF1A; IGF1R; RUNX1T1; CDK1; ACACA; HMOX1; ICAM1; MCL1; CCND2; IL2; CCNB1; SERPINE1; IFNG; IL4; IKBKG; CYP19A1; XIAP; PSMD3; SLC2A4; INSR; CD40LG; CYCS; CFLAR; AAGAB; INS; FCER2; IL13; MS4A2; ALPI; PSME3; G6PC1; APC; TRPM2; AKR1C3; SLC5A5; FXYD2; ALG5; STAT3; APP; EDN1; BIRC5; PGTS1; KCNH2; CHEK1; KCNMA1; CHRNA2; AKR1B1; XDH; EGFR; MMP2; MAPK1; IL10; IL6; TOP1; PCNA; ERBB2; CASP7; TYR; GSTP1; PTGES; NUF2; ADCY2; MET; DPEP1; DRD5; NQO1; AMY2A; PKIA; SLC6A2; ADRA2B; GABRA3; COL7A1; HTR1A; GABRA2; GABRA5; HTR2C; HTR1B; GABRA6; GABRG3; GABRE; VCAM1; FASN; EDNRA; CYP3A4; CYP1A2; MYC; CYP1A1; NR1I2; NPM1; ECE1; PARP4; CALCR; ITGB3; CTSB; MAPK8; CASP8; MMP3; FGF2; MMP10; IL1B; CREB1; SELE; PTGER3; PRKCG; ATF2; CSF2; PECAM1; MAPK8IP2; PTPN6; GAP43; DUOX2; INPPL1; LITAF; TNFSF6; CASP1; ENPP7; ADH1C; LYZ; ADRB1; LTA4H; MAOB; CTRB1; SOD1; TIMP1; TGFB1; COL1A1; COL3A1; ACTB; NPPA; NF1* |

Table S3 Top 50 KEGG pathways associated with the targets of selected compounds

| **KEGG pathways** |
| --- |
| *Pathways in cancer; Lipid and atherosclerosis; AGE-RAGE signaling pathway in diabetic complications; Human cytomegalovirus infection; Hepatitis B; TNF signaling pathway; Kaposi sarcoma-associated herpesvirus infection; Prostate cancer; Fluid shear stress and atherosclerosis; Endocrine resistance; Chemical carcinogenesis - receptor activation; IL-17 signaling pathway; PI3K-Akt signaling pathway; Human T-cell leukemia virus 1 infection; Small cell lung cancer; Epstein-Barr virus infection; p53 signaling pathway; HIF-1 signaling pathway; Apoptosis; Pancreatic cancer; Measles; Neuroactive ligand-receptor interaction; Hepatitis C; Proteoglycans in cancer; Toxoplasmosis; Relaxin signaling pathway; Bladder cancer; Platinum drug resistance; Colorectal cancer; Chagas disease; cGMP-PKG signaling pathway; Non-small cell lung cancer; Cellular senescence; Chronic myeloid leukemia; Breast cancer; Hepatocellular carcinoma; Estrogen signaling pathway; Viral carcinogenesis; cAMP signaling pathway; Calcium signaling pathway; FoxO signaling pathway; C-type lectin receptor signaling pathway; EGFR tyrosine kinase inhibitor resistance; T cell receptor signaling pathway; Th17 cell differentiation; Melanoma; Glioma; Apoptosis - multiple species; Pertussis; Leishmaniasis.* |

Table S4 the potential targets of SM in treating IPF

| **Target Gene Symbol** |
| --- |
| *STAT3; HIF1A; MMP3; PLAU; ESR1; TNF; TGFB1; VEGFA; IL6; ACTB; PTGS2; TP53; IL1B; IFNG; PIK3CG; IL4; IL13; SERPINE1; IL10; CDKN2A; MMP9; FGF2; EGFR; NR1I2; AKT1; CDKN1A; CCND1; MMP1; BCL2; PCNA; PTGS1; MAPK1; XIAP; JUN; CASP3; EDN1; CSF2; COL3A1; COL1A1; CFLAR; RELA; CCNA2 ;MAPK8 ;TIMP1; VCAM1; F10; ECE1; ERBB2; FOS; ALPI; AHSA1; MAPK14; MMP2; PIM1; NOS2; HMOX1; ICAM1; HTR2A; HSP90AB1; BIRC5; NR3C1; IL2; IGF1R; KCNMA1; LTA4H; PRSS1; AMY2A; CA2; SELE; PPARG.* |

Table S5 Compounds with the highest degree scores

| Compounds | Degree Value |
| --- | --- |
| apigenin | 79 |
| luteolin | 57 |
| ursolic acid | 54 |
| dihydroisotanshinoneⅠ | 43 |
| tanshinone iia | 41 |
| dan-shexinkum b | 40 |
| salviolone | 38 |
| dihydrotanshinlactone | 36 |
| 2-isopropyl-8-methylphenanthrene-3,4-dione | 33 |
| 4-methylenemiltirone | 33 |
| 1,2-DT-Quinone | 31 |
| isocryptotanshi-none | 31 |
| cryptotanshinone | 30 |
| 1,2,5,6-tetrahydrotanshinone | 29 |
| dan-shexinkum d | 29 |
| neocryptotanshinone ii | 29 |
